# Electro-steric opening of the clc-2 chloride channel gate

**DOI:** 10.1038/s41598-021-92247-3

**Published:** 2021-06-23

**Authors:** José J. De Jesús-Pérez, G. Arlette Méndez-Maldonado, Ana E. López-Romero, David Esparza-Jasso, Irma L. González-Hernández, Víctor De la Rosa, Roberto Gastélum-Garibaldi, Jorge E. Sánchez-Rodríguez, Jorge Arreola

**Affiliations:** 1 Physics Institute, Universidad Autónoma de San Luis Potosí, Ave. Dr. Manuel Nava #6, 78290 San Luis Potosí, SLP Mexico; 2 Departamento de Física, Centro Universitario de Ciencias Exactas e Ingenierías, Universidad de Guadalajara, Blvd. M. García Barragán #1421, 44430 Guadalajara, Jalisco Mexico; 3 CONACYT, School of Medicine, Universidad Autónoma de San Luis Potosí, Ave. V. Carranza 2005, Los Filtros, 78290 San Luis Potosí, SLP Mexico

**Keywords:** Biophysics, Physiology

## Abstract

The widely expressed two-pore homodimeric inward rectifier CLC-2 chloride channel regulates transepithelial chloride transport, extracellular chloride homeostasis, and neuronal excitability. Each pore is independently gated at hyperpolarized voltages by a conserved pore glutamate. Presumably, exiting chloride ions push glutamate outwardly while external protonation stabilizes it. To understand the mechanism of mouse CLC-2 opening we used homology modelling-guided structure–function analysis. Structural modelling suggests that glutamate E213 interacts with tyrosine Y561 to close a pore. Accordingly, Y561A and E213D mutants are activated at less hyperpolarized voltages, re-opened at depolarized voltages, and fast and common gating components are reduced. The double mutant cycle analysis showed that E213 and Y561 are energetically coupled to alter CLC-2 gating. In agreement, the anomalous mole fraction behaviour of the voltage dependence, measured by the voltage to induce half-open probability, was strongly altered in these mutants. Finally, cytosolic acidification or high extracellular chloride concentration, conditions that have little or no effect on WT CLC-2, induced reopening of Y561 mutants at positive voltages presumably by the inward opening of E213. We concluded that the CLC-2 gate is formed by Y561-E213 and that outward permeant anions open the gate by electrostatic and steric interactions.

## Introduction

Gating is a fundamental property whereby ion channels open a permeation pathway so that ions can passively flow through membranes, ensuring electric communication^1–4^. In some channels, a sudden change in the membrane potential, ligand binding or lipid bilayer deformation, triggers a propagating cascade of structural rearrangements that opens the permeation pathway^1–7^. Alternatively, gating could rely on the permeant ion as reported for K^+^ and Cl^−^ channels^8^. This hypothesis suggests that ion permeation is coupled to channel gating, a mechanism that has been proposed to operate in channels and transporters^9^.

The Cl^−^ channels (CLC-0, CLC-1, CLC-2, CLC-Ka, and CLC-Kb) and Cl^−^/H^+^ exchangers of the CLC protein family are structurally conserved^10^. All are dimers harbouring a pore in each monomer that is controlled by a highly conserved glutamate residue known as glutamate gate located inside the pore^11–14^. Since CLC channels are gated by voltage despite lacking a canonical voltage sensor^10^, the pore structure suggests that gating may occur unconventionally^8^. The glutamate gate could be opened by repulsion or by protonation when voltage drives a permeant anion or a proton into the pore^12,15–22^. Gating by permeant anions has been proposed for CLC-0 and CLC-1 channels^18,19,23,24^. However, the proton sensitivity analysis of a conservative mutation at the glutamate gate, suggested that protonation of this residue by intracellular protons is the major voltage-dependent step in CLC-0 gating ^21,25^, a process catalysed by extracellular Cl^−^ anions^26^. CLC-2 gating deviates from the latter scenario; it relies on hyperpolarization and intracellular Cl^−^ while depolarization causes deactivation^15,27,28^. Moreover, voltage-dependent gating can occur even with impermeable anions present in the cytosolic side^17^. Remarkably, intracellular acidification has little or no effect on gating whereas extracellular acidification increases the open probability^29^. Still, extracellular protonation cannot explain CLC-2 activation because gating happens under unfavourable protonation conditions^16,17^. Given these observations, we proposed that hyperpolarization drives Cl^−^ inside the pore causing E213 to open by electrostatic and steric repulsion^15,17^, then extracellular protonation of E213 residue stabilizes the open conformation^16^. However, the molecular details supporting this mechanism are still waiting to be described and evidence supporting the hypothesis is still scarce.

In this work, we performed a functional analysis combined with site-directed mutagenesis of critical residues for gating located within the pore to determine the molecular entities involved in the opening of the pore gate in CLC-2, a Cl^−^ channel highly expressed throughout the central nervous system that controls chloride transport, extracellular chloride homeostasis, neuronal excitability, aldosterone secretion and heart rate^10,28,30^.

## Results

### The homology structure of CLC-2

**Figure 1:**
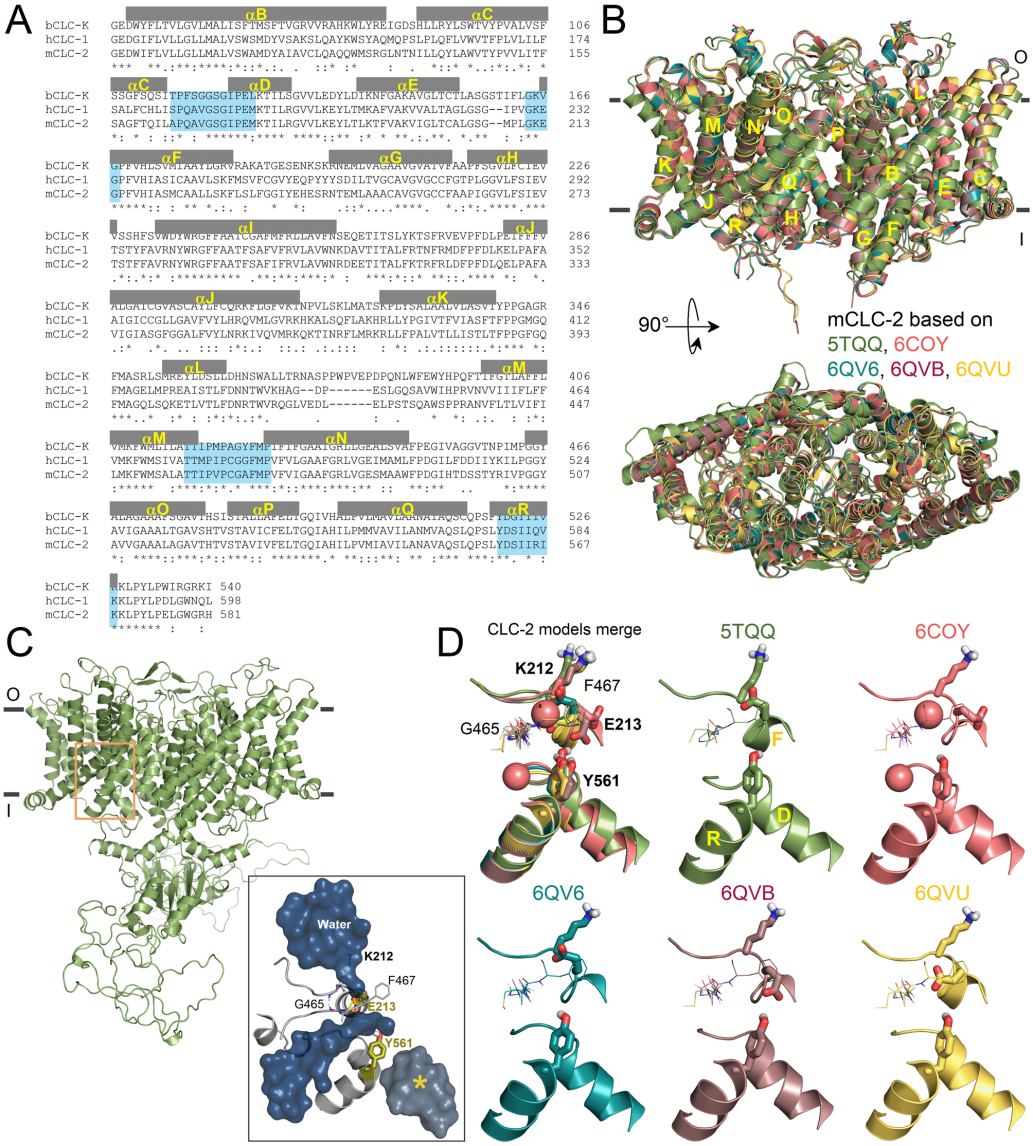
Homology models of the CLC-2 structure. **(A)** Sequence alignment of the transmembrane region of bovine CLC-K (bCLC-K), human CLC-1 (hCLC-1), and mouse CLC-2 (mCLC-2). Residues forming B-R alpha helices are shown in grey and pore region residues are highlighted in blue. **(B)** Structural alignment of the homology models for the CLC-2 structure. Homology structures were built using the cryo-EM structure of CLC-K (5TQQ, green) and hCLC-1 (6COY, salmon; 6QV6, cyan; 6QVB, redwood; 6QVU, yellow) channels as templates. Views of the transmembrane domains (yellow letters) perpendicular to membrane plane (above) and from the top (below). Parallel grey lines indicate external (o) and internal (i) membrane limits. The RMSD of backbone atoms were < 0.31 Å and the C-score = 1.98 calculated by I-Tasser (2 is the upper limit). **(C)** mCLC-2^CLC-K^ model structure showing the transmembrane and intracellular cystathionine-β-synthase (CBS) domains. The external and internal membrane limits are indicated by parallel grey lines. The orange square indicates the intracellular pore region shown in **(D)**. Grey square shows the canonical pore flooded with water represented as a dark surface. The intracellular alternative pathway, unconnected to the canonical pore, is shown in grey marked with a yellow asterisk. Y561 and E213 are in olive. **(D)** Pore region of mCLC-2 models. Pore regions superposition (CLC-2 models merge) from homology structures. Sticks represent K212, E213, and Y561. The sidechain of E213 adopted different positions depending on the template; away from Y561 in 5TQQ, 6COY, and 6QV6 based models and closer to Y561 in 6QVB and 6QVU based models. Chloride ions (pink spheres) from hCLC-1 6COY were placed in the 6COY-based CLC-2 model.

The structure of the CLC-2 chloride channel remains unsolved. In this work, we utilized homology modelling to obtain model structures of the mouse CLC-2. We constructed CLC-2^CLC-K^ based on bovine CLC-K structure (PDB: 5TQQ^14^), and CLC-2^CLC-1^ based on human CLC-1 structures (PDBs: 6COY, 6QVB, 6QV6, 6QVU^13,31^) available later. mCLC-2 is 48.77% identical to CLC-K within the transmembrane domain (TMD; Fig. 1A). The pore regions are 63.89% identical, but a valine replaces the glutamate gate in CLC-K. Compared to hCLC-1, CLC-2 is 67.98% identical within TMD and 83.33% within the pore region (Fig. 1A, blue region). CLC-2^CLC-K^ and CLC-2^CLC-1^ homodimers are rhombus-shaped with a TMD comprising 17 α-helices (termed B-R) (Fig. 1B). Helix A (residues 1 to 96), which is not in the template structures, could not be modelled. Only CLC-2^CLC-K^ included the C-terminus with two cystathionine-β-synthase (CBS) domains (Fig. 1C). Interestingly, this homology structure showed the presence of an alternative water-filled cavity towards the intracellular side (indicated by the yellow asterisk in the grey square in Fig. 1C). However, it seems to be isolated from the canonical pore (indicated by dark blue in the inset in Fig. 1C).

From these models, we inferred that the canonical CLC-2 pore (orange box in Fig. 1C) is formed by residues 164APQAVGSGIPEM175, 211GKEG214, 458TTIPVPCGAFMP469, and 561YDSIIRIK568 located at the N-termini of αD, αF, αN, and αR helices, respectively. E213 adopts different rotational angles depending on the template (Fig. 1D). While E213 in the CLC-2^CLC-K^ (green) or 6QV6-based CLC-2^CLC-1^ (cyan) is directed to the extracellular side, in the 6COY-based CLC-2^CLC-1^ (salmon) E213 pointed to F helix allowing occupation of the pore by two Cl^−^ ions. In the 6QVB-based CLC-2^CLC-1^ (redwood) E213 pointed to Y561 and was in close proximity with the backbone of 465GAF467 in 6QVU-based CLC-2^CLC-1^ (yellow).

### Y561 and E213 are coupled to control CLC-2 gating

**Figure 2:**
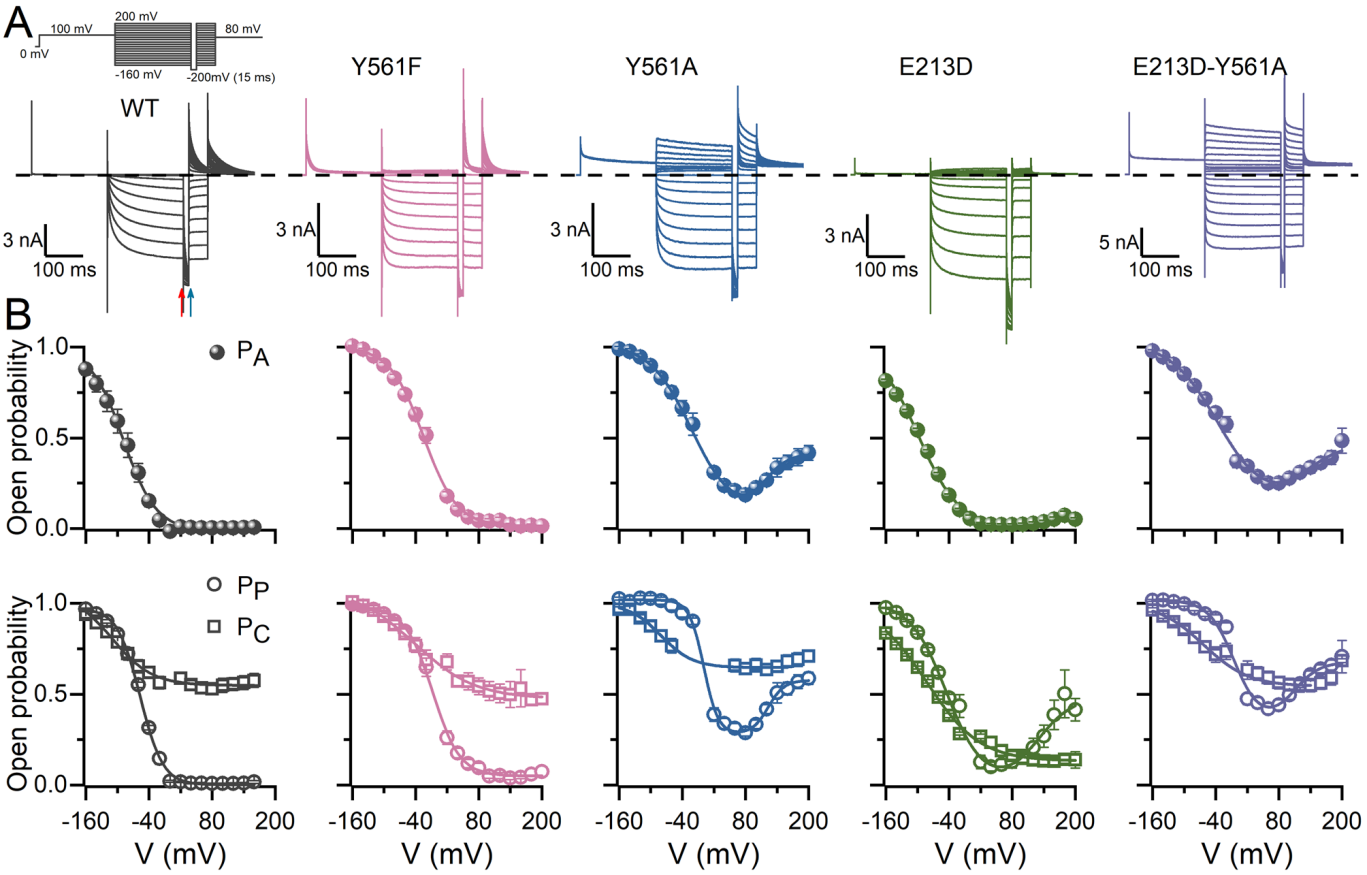
Voltage-dependent activation of CLC-2 depends on both Y561 and E213. **(A)** Colour coded Cl^−^ currents recorded from five different HEK293 cells expressing WT CLC-2, Y561F, Y561A, E213D, and E213D-Y561A channels. Cl^−^ currents were elicited by the voltage protocol shown in the upper left corner. The protocol consisted of voltage steps from − 160 or − 200 to + 200 mV in 20 mV increments and a repolarization voltage to + 80 mV to record tail currents. We intercalated a 15 ms/− 200 mV step and used the magnitude of the currents measured before and after the interpulse (red and blue arrows, respectively) to calculate the open probability of pore (P_P_) and common (P_C_) gates (see methods). Cl^−^ currents were recorded using pH_i_ = pH_o_ = 7.3 and [Cl^−^]_i_ = [Cl^−^]_o_ = 140 mM. **(B)** The voltage dependency of the apparent open probability (P_A_, spheres), pore (P_P_, circles) and common (P_C_, squares) gates computed for WT CLC-2, Y561F, Y561A, E213D, and E213D-Y561A channels. Upper row: P_A_; lower row: P_P_ and P_C_. Continuous lines are fits with a single (P_A_ of WT, Y561F, and E213D; P_P_ of WT and Y561F; P_C_ of all channels) or double (P_A_ of Y561A and E213D-Y561A; P_P_ of Y561A, E213D, and E213D-Y561A) Boltzmann equation to determine voltage-dependent parameters V_0.5_ and z listed in Table 1.

Some CLC-2 homology models showed E213 oriented away from Y561 suggesting that Y561 is unnecessary for gating, an idea echoed by a recent computational work aimed at determining CLC-2 fast gating^32^. However, in the CLC-2 6QVB model, E213 is facing Y561. This configuration is similar to that proposed for CLC-1 where the central Y578 residue participates in common gating^33^. These observations led us to consider that E213 could interact with Y561 to control CLC-2 gating. To assess this idea, we produced single and double mutant channels replacing Y561 residue either by F or A, and the E213 residue by D to maintain the negative charge at this position but with a shortened side chain. The activity of wild type (WT) and mutant channels was measured using both patch-clamp and cut-open oocyte voltage-clamp. Figure 2A shows representative currents from WT, Y561F, Y561A, E213D, and E213D-Y561A channels using the voltage protocol depicted in the upper left part of the figure. Y561F channel presented a similar inwardly rectifying activation as the WT channel but showed faster activation kinetics. In contrast, the Y561A channel presented both inward and outward currents, supporting the idea that this residue is important for maintaining the closed state. Remarkably, voltage-activated currents through the Y561A channel were observed at positive and negative potentials. The currents activated fast but displayed a slow decay at positive voltages. If E213 interacts with Y561, then a residue with a shorter side chain at position 213 would disturb this interaction and affect the activation of the channel similarly. Indeed, E213D mutant channel displayed both inward and outward rectifying currents. The inward currents were like those observed in the WT channel whereas the outward currents exhibited a slow time course, tail currents were not observed at + 80 mV. The double mutant E213D-Y561A had currents like those of Y561A but with a larger instantaneous component.

Plots in the upper row of Fig. 2B show the voltage dependence of the apparent open probability (P_A_) for WT, Y561F, Y561A, E213D, and E213D-Y561A channels. The plots were fitted with a single or double Boltzmann function (Eq. 3) to determine the half-maximum activation voltage (V_0.5_, in mV) and the apparent charge (z) quantifying their voltage dependence. The V_0.5_ values of Y561F, Y561A and E213D-Y561A mutants were rightward shifted (> + 54 mV) relative to WT whereas z remained between -0.84 (WT) and -0.60 (E213D-Y561A). P_A_ of both Y561A and E213D-Y561A reached a minimum value of 0.2 at around + 80 mV and then increased to about 0.4 at + 200 mV, a behaviour we referred to as re-opening (Fig. 2B, Table 1). Moreover, we determined that this reopening behaviour corresponds to currents flowing through CLC-2 channels since 5 mM Zn^2+^, a blocker of CLC-2^34^, effectively blocked both positive and negative currents of the Y561A channel (Supplementary Fig. 1). In contrast, V_0.5_/z values of E213D were like those of WT: − 92.4 ± 1.4 mV/− 0.60 ± 0.02 vs − 89.3 ± 8.5 mV/− 0.84 ± 0.04, respectively, and P_A_ displayed a less pronounced re-opening at positive potentials. The voltage dependence of the apparent open probability of the pore (P_P_) and common (P_C_) gates of WT, Y561F, Y561A, E213D, and E213D-Y561A channels are shown in the lower row of Fig. 2B. Notably, in the Y561A, E213D, and E213D-Y561A channels, P_P_ increased at positive potentials. Since P_C_ remains partially open, the re-opening behaviour of P_P_ explains the outward currents at positive voltages in these channels. Table 1 summarizes the V_0.5_ and z values of P_p_ and P_C_ calculated from single or double Boltzmann fits (continuous lines) to the data. Like P_A_, the V_0.5_ values for P_P_ of Y561F, Y561A, and E213D-Y561A were rightward shifted by >  + 40 mV relative to WT without changing z.

**Table 1:** Effect of [H^+^]_i_ and [Cl^−^]_O_ on the voltage-dependent parameters of WT and mutants CLC-2 channels.

Channel		${V}_{0.5}^{{P}_{A}}$ (mV)	${z}_{{P}_{A}}$	${V}_{0.5}^{{P}_{P}}$ (mV)	${z}_{{P}_{P}}$	${V}_{0.5}^{{P}_{C}}$ (mV)	${z}_{{P}_{C}}$	Er (mV)	pH_i_	[Cl^−^]_o_ (mM)	n
*WT* WT-OOC		*− 89.3* ± *8.5* *− *96.9 ± 7.5	*− 0.84* ± *0.04* *− *0.87 ± 0.04	*− 56.6* ± *1.0* *− *72.3 ± 1.4	*− 1.17* ± *0.07* *− *1.24 ± 0.10	*− 110.9* ± *6.3* *− *115.1 ± 5.5	*− 0.75* ± *0.03* *− *1.17 ± 0.13	*1.9* ± *0.3*	*7.3* 7.3	*140* 140	*5* 7
WT		*− *79.1 ± 5.8	*− *0.78 ± 0.02	*− *39.5 ± 2.2	*− *1.19 ± 0.07	*− *82.9 ± 6.1	*− *0.54 ± 0.04	57.4 ± 0.6	7.3	10	5
*Y561F* Y561F-OOC		*− 34.8* ± 9.4* *− *29.7 ± 7.6	*− 0.8* ± *0.0* *− *0.57 ± 0.05	*− 13.8* ± 6.4* *− *5.66 ± 9.9	*− 1.1* ± *0.1* *− *0.71 ± 0.05	*− 51.2* ± *6.4* *− *37.3 ± 17.9	*− 0.4* ± *0.0* *− *0.62 ± 0.1		*7.3* 7.3	*140* 140	*9* 7
Y561F		*− *68.9 ± 5.4^†^	*− *0.67 ± 0.02	*− *6.3 ± 4.4	*− *0.73 ± 0.04^†^	*− *71.9 ± 4.5	*− *0.62 ± 0.07	52.5 ± 1.6	7.3	10	6
Y561F	O	*− *36.0 ± 5.1	*− *0.68 ± 0.03	*− *19.7 ± 2.1	*− *0.95 ± 0.08	*− *36.1 ± 7.9	*− *0.53 ± 0.06		5.5	140	8
	rO	172.1 ± 9.9	0.76 ± 0.10	188.0 ± 6.4	*− *0.71 ± 0.10						
Y561F	O	*− *58.6 ± 18.8	*− *0.69 ± 0.09	*− *21.1 ± 8.6	*− *1.09 ± 0.17	*− *78.7 ± 23.2	*− *0.63 ± 0.06		4.3	140	5
	rO	163.3 ± 7.2	0.98 ± 0.14	171.0 ± 12.2	0.83 ± 0.11						
Y561A	O	*− *21.2 ± 6.2*	*− *0.79 ± 0.07	5.1 ± 2.8 *	*− *1.25 ± 0.02	*− *65.6 ± 10.6	*− *0.81 ± 0.06		7.3	140	7
	rO	130.8 ± 3.3	1.20 ± 0.08	123.3 ± 3.7	1.28 ± 0.07						
Y561A-OOC	O	*− *37.1 ± 3.6	*− *0.77 ± 0.02	*− *16.7 ± 9.6	*− *0.85 ± 0.05	*− *64.0 ± 1.88	*− *1.05 ± 0.01		7.3	140	3
	rO	89.4 ± 5.5	1.23 ± 0.07	82.0 ± 5.3	1.17 ± 0.03						
Y561A	O	*− *58.7 ± 9.2^‡^	*− *0.58 ± 0.01	27.2 ± 3.8^‡^	*− *1.02 ± 0.02	*− *74.5 ± 7.3	*− *0.64 ± 0.07	57.5 ± 0.8	7.3	10	6
	rO	193.0 ± 4.1^‡^	0.89 ± 0.10	147.0 ± 21.6	0.56 ± 0.06^‡^						
Y561A	O	*− *32.6 ± 5.3	*− *0.75 ± 0.06	*− *9.0 ± 4.2	*− *0.93 ± 0.06	*− *77.2 ± 15.9	*− *0.98 ± 0.16		5.5	140	9
	rO	137.8 ± 11.6	0.93 ± 0.10	111.5 ± 12.46	*− *0.77 ± 0.08^‡^						
Y561A	O	*− *36.5 ± 7.6	*− *0.73 ± 0.08	*− *14.3 ± 5.1^‡^	*− *0.96 ± 0.10	*− *66.7 ± 12.1	*− *0.77 ± 0.07		4.3	140	10
	rO	126.0 ± 5.6	0.63 ± 0.05	100.4 ± 6.5	0.84 ± 0.08^‡^						
E213D	O	*− *92.4 ± 1.4	*− *0.60 ± 0.02	*− *43.0 ± 2.8	*− *0.74 ± 0.03	*− *86.0 ± 6.1	*− *0.51 ± 0.01		7.3	140	8
	rO	194.6 ± 18.2	0.42 ± 0.03	209.0 ± 19.6	0.55 ± 0.06						
E213D-Y561A	O	*− *35.4 ± 4.1*	*− *0.67 ± 0.03	*− *5.6 ± 1.5*	*− *1.11 ± 0.10	*− *63.3 ± 9.4	*− *0.61 ± 0.05		7.3	140	7
	rO	122.9 ± 4.7	1.09 ± 0.09	101.5 ± 6.7	1.06 ± 0.06						

${\mathrm{V}}_{0.5}^{{\mathrm{P}}_{\mathrm{A}}}$ = voltage to reach an open probability of 0.5, ${\mathrm{z}}_{{\mathrm{P}}_{\mathrm{A}}}$ = apparent charge of the open-closed transition, ${\mathrm{V}}_{0.5}^{{\mathrm{P}}_{\mathrm{P}}}$ = voltage to reach a 0.5 open probability of Glu_gate_, ${\mathrm{z}}_{{\mathrm{P}}_{\mathrm{P}}}$ = apparent charge of the Glu_gate_, ${\mathrm{V}}_{0.5}^{{\mathrm{P}}_{\mathrm{C}}}$ = voltage to reach a 0.5 open probability of the common gate, ${\mathrm{z}}_{{\mathrm{P}}_{\mathrm{C}}}$ = apparent charge the common gate. These parameter values were calculated by fitting the data to the Boltzmann equation (Eq. 3). Er = reversal potentials determined by current–voltage relationship interpolations. Data were collected from HEK 293 cells and from cut-open oocytes (OOC). Within a column, those mean values labelled with *, †, or ‡ were statistically different to that of to WT, Y561A, or Y561F, respectively. Reference values of to WT, Y561A, and Y561F were calculated from recording obtained under control conditions (pH_i_ = pH_e_ = 7.3 and [Cl^−^]_i_ = [Cl^−^]_e_ = 140 mM). Mean values were compared using a one-way ANOVA with a Tukey post hoc test with p < 0.01.

In addition to the changes in voltage dependence and magnitude of P_P_ above described the voltage dependence of P_C_ of Y561F, Y561A, E213D, and E213D-Y561A mutant channels was also shifted to the right by + 60, + 45, + 25, and + 48 mV, respectively. P_C_ of the Y561A channel was slightly higher than that for WT, but it was close to zero in the E213D mutant. Similar results were obtained using the cut-open oocyte technique with the WT, Y561F, and Y561A channels (Supplementary Fig. 2 and Table 1).

**Figure 3:**
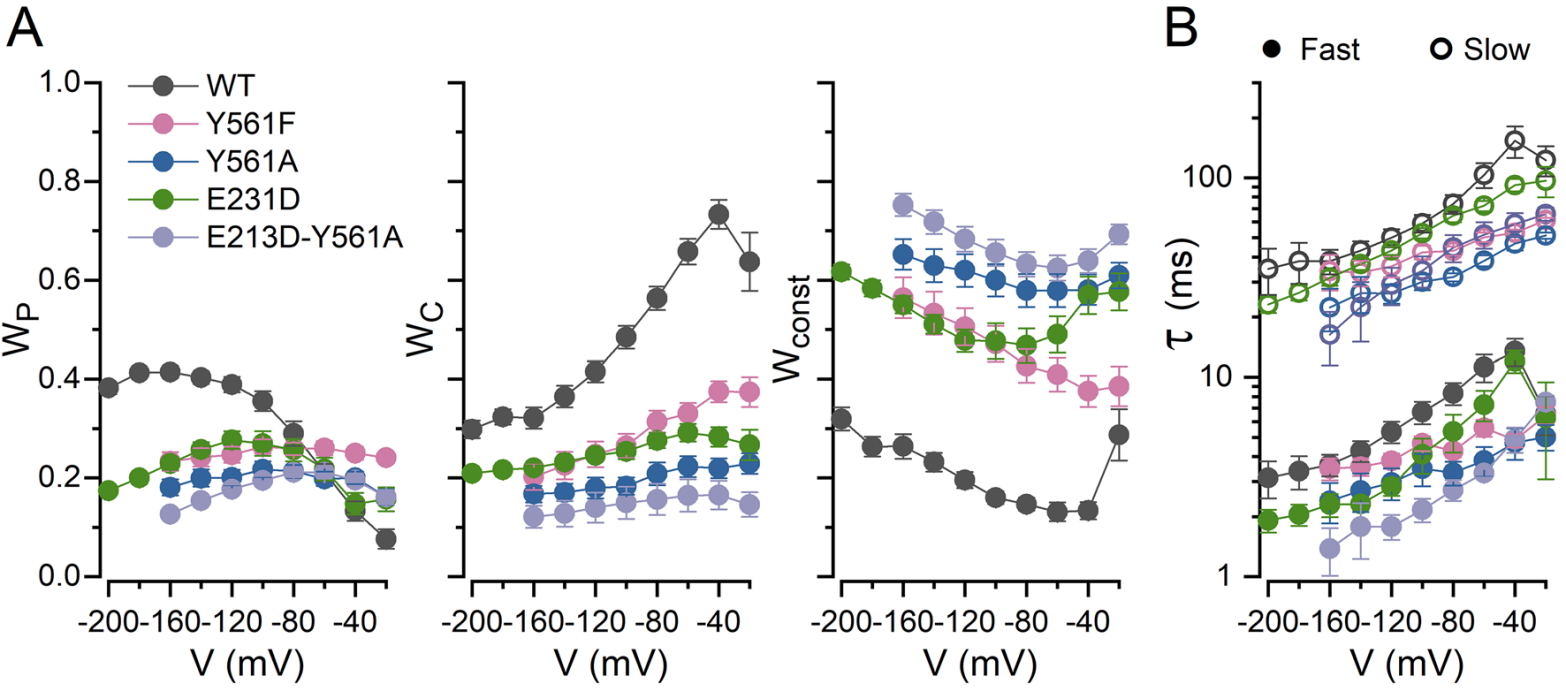
Kinetics analysis of WT and Y561 and E213 mutant channels. **(A)** The relative contribution of fast (W_P_), slow (W_C_) and constant (W_const_) components of the total Cl^−^ current generated by the colour coded indicated channels. **(B)** Voltage dependence of fast (τ_f_, closed circles) and slow (τ_s_, open circles) time constants of the Cl^−^ currents generated by the channels indicated in **(A)**. Whole cell Cl^−^ currents were fitted with a biexponential function (Eq. 2) to determine W_p_, W_C_, W_const_, τ_f_, and τ_s_.

The above data suggested that these mutations were altering both the pore and the common gates of CLC-2. We corroborated this idea by quantifying the fractional contribution of the fast (W_P_), slow (W_c_) and constant (W_const_) components of the whole cell Cl^−^ current, as well as their time constants (τ_f_ and τ_s_) using a biexponential curve fit (Eq. 2) to the current recordings. Figure 3A shows the contribution of W_P_ (left), W_C_ (middle) and W_const_ (right) to the whole cell currents generated by WT, Y561F, Y561A, E213D, and E213D-Y561A channels. W_P_ associated with the fast gating decreased at negative potentials, whereas W_C_ associated with the common gating decreased in the mutants at all voltages. W_const_ increased at all potentials in all mutants indicating that the open probability of the channels increased at all potentials. Figure 3B shows the voltage dependence of the fast (closed symbols) and slow (open symbols) time constants for the currents generated by the WT, Y561F, Y561A, E213D, and E213D-Y561A channels. The fast time constants were between 1.5 and 10 ms whereas the slow time constants were between 20 and 100 ms. Both time constants increased at depolarized voltages (Fig. 3B).

### The double mutant cycle analysis and anomalous mole fraction behaviour reveal the interaction of Y561 and E213 in CLC-2

The previous data show that Y561 together with E213 keep the pore of CLC-2 closed. Furthermore, when Y561 is mutated, the common gate opening is facilitated and E213 can move in both outward and inward directions as indicated by the re-opening. These findings suggested that E213 and Y561 are interacting. Evidence for this idea was obtained by performing a double mutant cycle analysis^35^ and by determining the anomalous mole fraction (AMF) behaviour.

**Figure 4:**
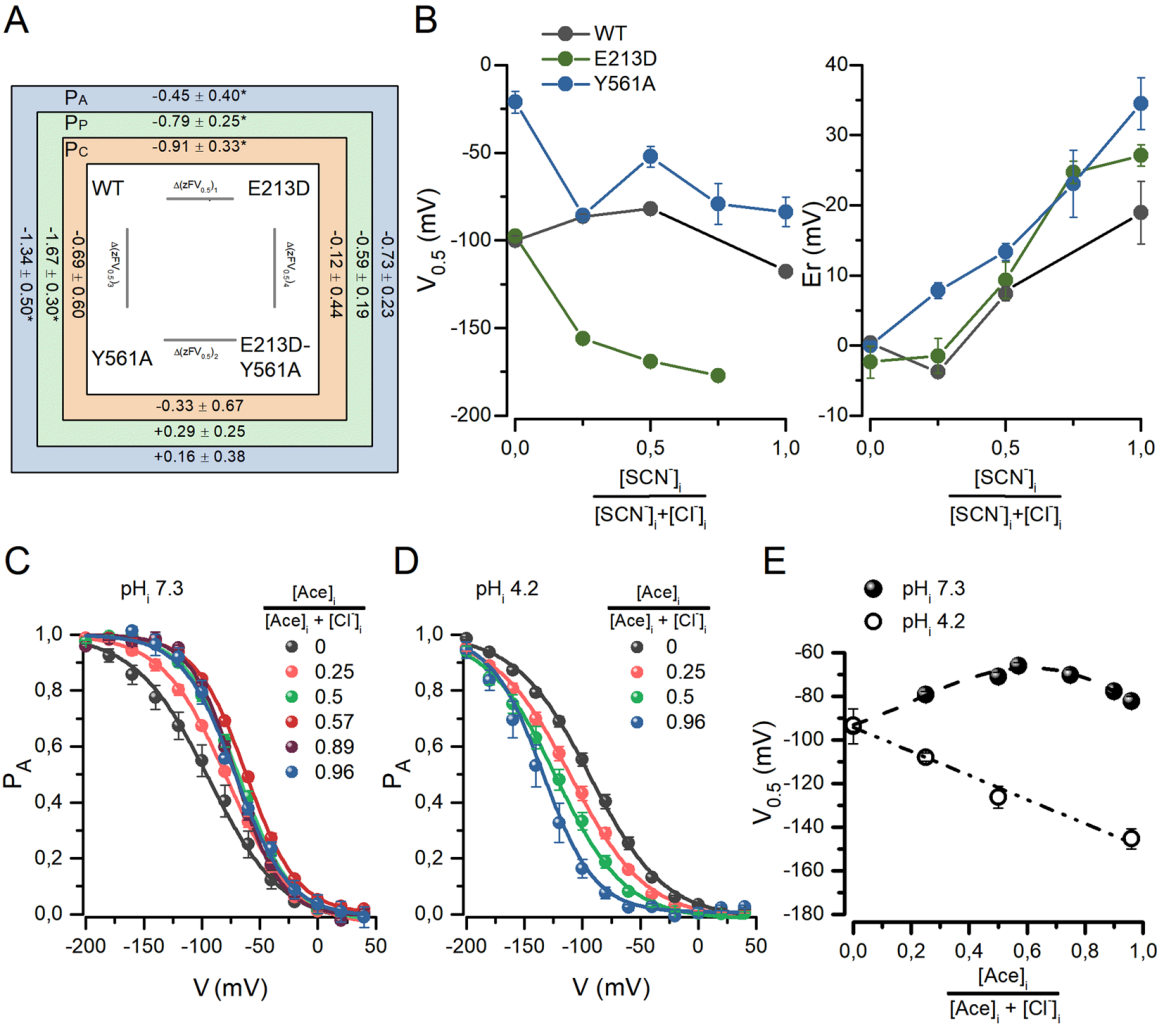
Double mutant cycle analysis and anomalous mole fraction behaviour show coupling of E213 and Y561. **(A)** Double mutant cycle analysis. The analysis was performed using the z and V_0.5_ obtained for P_A_ (blue square), P_P_ (green square), and P_C_ (orange square) for the WT, E213D, Y56A and E213D-Y561A channels listed in Table 1. Δ(zFV_0.5_)_1_, Δ(zFV_0.5_)_2_, Δ(zFV_0.5_)_3_, and Δ(zFV_0.5_)_4_ are the energy change in the voltage-dependent gating caused by a given mutation calculated using Eq. (1). For example, Δ(zFV_0.5_)_1_ is the energy change induced by mutating E213 in the WT CLC-2. The listed energy change values are in kCal/mol. The asterisks indicate statistically significant different pair Δ(zFV_0.5_)_1_:Δ(zFV_0.5_)_2_ and Δ(zFV_0.5_)_3_:Δ(zFV_0.5_)_4_ values. **(B)** Anomalous mole fraction behaviour of the voltage-dependent activation and reversal potential of WT (n = 5–9), E213D (n = 6–10), and Y561A (n = 4–7) channels. AMF behaviour was evaluated using SCN^−^/Cl^−^ mixtures. V_0.5_ values were calculated using the Boltzmann equation to fit the negative portion of the curves whereas Er was calculated by interpolation in the current–voltage relationships. At SCN^−^ mole fraction = 1, we could not determine E213D V_0.5_. **(C,D)** Voltage-dependent activation determined at pHi of 7.3 (n = 5–10) and 4.2 (n = 5–7) using different acetate mole fractions. P_A_ was calculated using the tail current magnitude. The resulting curves were normalized to their respective tail current maximum obtained after fitting curves with the Boltzmann equation. Continuous lines are fits from which the voltage-dependent parameters V_0.5_ and z were computed. **(E)** V_0.5_ plotted against acetate mole fractions. Filled symbols correspond to data obtained at pH_i_ 7.3 whereas open symbols were collected at pH_i_ 4.2. V_0.5_ values were obtained from Boltzmann fits to data shown in panels C and D.

Double-mutant cycle analysis has been extensively applied to determine the interaction and its strength between pairwise residues^36,37^. We applied this thermodynamic analysis to determine whether E213 and Y561 are energetically coupled in the gating process of CLC-2. In the square in Fig. 4A, Δ(zFV_0.5_)_1_, Δ(zFV_0.5_)_2_, Δ(zFV_0.5_)_3_, and Δ(zFV_0.5_)_4_ are the energy change (in kCal/mol) in gating caused by a given mutation. These energy changes were calculated^37^ as:

1 $$ \Delta \left( {zFV_{{0.5}} } \right) = ~ - F\left( {z_{{WT}} V_{{0.5,WT}}  - ~z_{{mut}} V_{{0.5,mut}} } \right) $$

where *z*_*WT*_, *z*_*mut*_, *V*_*0.5,WT*_ and *V*_*0.5,mut*_ are the apparent charges and the half-maximum activating voltages of the WT and mutant channels listed in Table 1. This analysis was performed for triplicated using the z and V_0.5_ values determined at negative voltages for P_A_, P_P_, or P_C_ of WT, Y561A, E213D, and E213D-Y561A channels (colour coded in Fig. 4A). The mean (± SD) values for Δ(zFV_0.5_)_1_, Δ(zFV_0.5_)_2_, Δ(zFV_0.5_)_3_, and Δ(zFV_0.5_)_4_ are shown. To infer whether E213 and Y561 are coupled we compared the energy changes induced by mutating E213 in the WT and the Y561A mutant channel. If mutating E213 induces the same change on the voltage dependence of WT and Y561A, then the energy changes should be the same regardless of the background used to make the mutation, that is Δ(zFV_0.5_)_1_ = Δ(zFV_0.5_)_2_. However, if the voltage dependence of gating is altered differently in WT and Y561A, then Δ(zFV_0.5_)_1_ will be different to Δ(zFV_0.5_)_2_ implying that E213 and Y561 are coupled^37^. We statistically compared the pair values Δ(zFV_0.5_)_1_:Δ(zFV_0.5_)_2_ and Δ(zFV_0.5_)_3_:Δ(zFV_0.5_)_4_ listed in Fig. 4A. Of these pairs, only Δ(zFV_0.5_)_3_ and Δ(zFV_0.5_)_4_ within the P_C_ cycle were not statistically different. In addition, using the Δ(zFV_0.5_)_1_ and Δ(zFV_0.5_)_2_ values calculated using the voltage-dependent parameters of P_P_ (green square), we obtained that the Δ(zFV_0.5_)_2_ − Δ(zFV_0.5_)_1_ =  + 1.08 kCal/mol, a value that is different from 0. Overall, this analysis indicates that Y561 and E213 are energetically coupled.

We previously showed that the voltage dependence of CLC-2, measured by V_0.5_, displays AMF behaviour in cells dialyzed with SCN^−^ mole fractions^15^. Hence, if the anions are disturbing the Y561-E213 interaction responsible for voltage dependence gating of CLC-2 then the AMF behaviour of the voltage dependence will be altered in channels with these mutated residues. We determined the V_0.5_ of the WT, E213D, and Y561A channels exposed to SCN^−^/Cl^−^ mixtures from the cytosolic side. Figure 4B shows the relationship between V_0.5_ and SCN^−^ mole fractions. The voltage dependence of the WT channel (black) displayed a concave relationship. However, the AMF behaviour of E213D V_0.5_ (4B, green) was absent; as the SCN^−^ mole fraction increased, V_0.5_ became more negative, albeit is a non-linear fashion. Y561A (Fig. 4B, blue), in contrast, showed V_0.5_ values that tended to be less negative than those determined for WT channels. However, in both mutants, the reversal potential increased as SCN^−^ mole fraction increased; with SCN^−^ mole fraction = 1, the reversal potentials of these mutant currents were more positive than that of WT (Fig. 4B, right) suggesting that the SCN^−^ permeability is altered. The single-channel Cl^−^ current of Y561A determined by noise analysis (− 0.26 ± 0.01 pA at − 100 mV, n = 6) was similar to WT (0.23 ± 0.02 pA at + 100 mV, n = 5) and the single-channel current of human CLC-2 at − 100 mV (− 0.23 ± 0.02 pA)^38^ indicating that the single-channel conductance is not altered in this mutant. The AMF behaviour of WT CLC-2 was also observed with internal solutions containing acetate/Cl^−^ mole-fractions at pH 7.3 (Fig. 4C,E). Then, we reduced the acetate negative charge by lowering the internal solutions pH to 4.2 (pK of acetate = 4.75; uncharged fraction = 0.22) to study its effect on the voltage gating. Under this condition, increasing the acetate mole fraction shifted the voltage-dependent activation curves towards negative voltages (Fig. 4D). V_0.5_ had a linear relationship with acetate/Cl^−^ mole-fractions (Fig. 4E, open circles) as in cells dialyzed only with Cl^−^^15^. Together these data support the idea that CLC-2 voltage gating is controlled by coupled Y561-E213 forming the gate that is opened by repulsion when the permeant anions occupy the pore.

### Mutating Y561 renders sensitivity to extracellular Cl^−^ and intracellular H^+^

**Figure 5:**
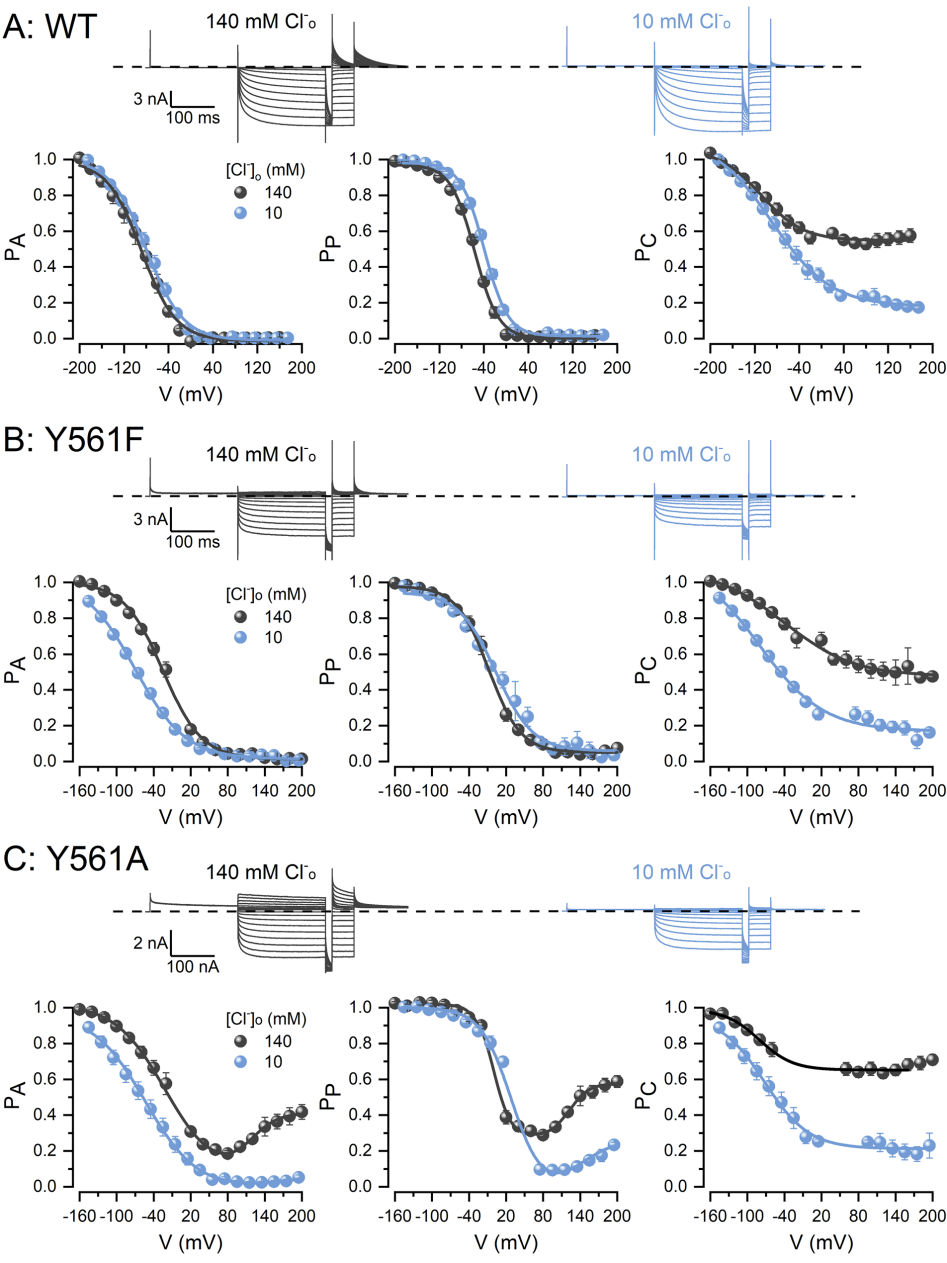
Y561F and Y561A CLC-2 mutant channels are sensitive to extracellular chloride. **(A–C)** Top panels: Cl^−^ currents recorded from HEK293 cells expressing WT CLC-2 **(A)**, Y561F **(B)**, or Y561A **(C)** exposed first to 140 (grey) and then 10 (blue) mM [Cl^−^]_o_. [Cl^−^]_i_ = 140 mM and pH_i_ = pH_o_ = 7.3. Channels were activated using the voltage protocol shown in Fig. 2A. **(A–C)** Bottom panels: Voltage dependence of P_A_ (left), P_P_ (middle), and P_C_ (right) for WT CLC-2 **(A)**, Y561F **(B)** and Y561A **(C)** determined first with 140 (grey) and then with 10 (blue) mM [Cl^−^]_o_. [Cl^−^]_i_ = 140 mM and pH_i_ = pH_o_ = 7.3. Lines are fits of the data with a single (**A,B**, and P_A_ from 10 mM Cl^−^ data and P_C_ in **C**) or double (P_A_ from 140 mM Cl^−^ data and P_P_ in **C**) Boltzmann equation. Voltage-dependent parameters V_0.5_ and z are listed in Table 1.

A hallmark of CLC-2 gating is its remarkably low sensitivity to extracellular [Cl^−^] ([Cl^−^]_o_) and intracellular [H^+^] ([H^+^]_i_)^15^. The re-opening observed at the positive voltage in the Y561A mutant channel suggested that gating occurred at both negative and positive voltages and that E213 is unconstrained and able to swing in the outward and inward directions. We wondered if Cl^−^ influx would knock-in E213, just like the Cl^−^ efflux knocks-out the E213 gate in the WT CLC-2 channel, thus inducing E213 re-opening at positive potentials. To assess this idea, we lowered the [Cl^−^]_o_ from 140 to 10 mM. Decreasing the Cl^−^ influx had little effect on the kinetics of WT, Y561F and Y561A currents (black vs. blue traces in Fig. 5A–C). In the WT channel, the voltage dependence of P_A_ and P_P_ were not affected (Fig. 5A, Table 1). However, in Y561F mutant channels (Fig. 5B), the V_0.5_ of P_A_ was shifted by − 34 mV, whereas the effect on P_P_ was negligible (Table 1). Interestingly, the reopening previously observed in the Y561A mutant channel was abolished by lowering the extracellular Cl^−^ (Fig. 5C). P_A_ was shifted by about -37 mV and P_P_ diminished from 0.3 to 0.1 at positive voltages. This indicates that decreasing the Cl^−^ influx diminishes the re-open probability of E213 at positive potentials in the Y561A mutant channel, suggesting that Y561 participates in the stability of the closed state through E213. Under low [Cl^−^]_o_ conditions, P_C_ was nearly equally diminished with little effects on V_0.5_ in all channels (Table 1).

**Figure 6:**
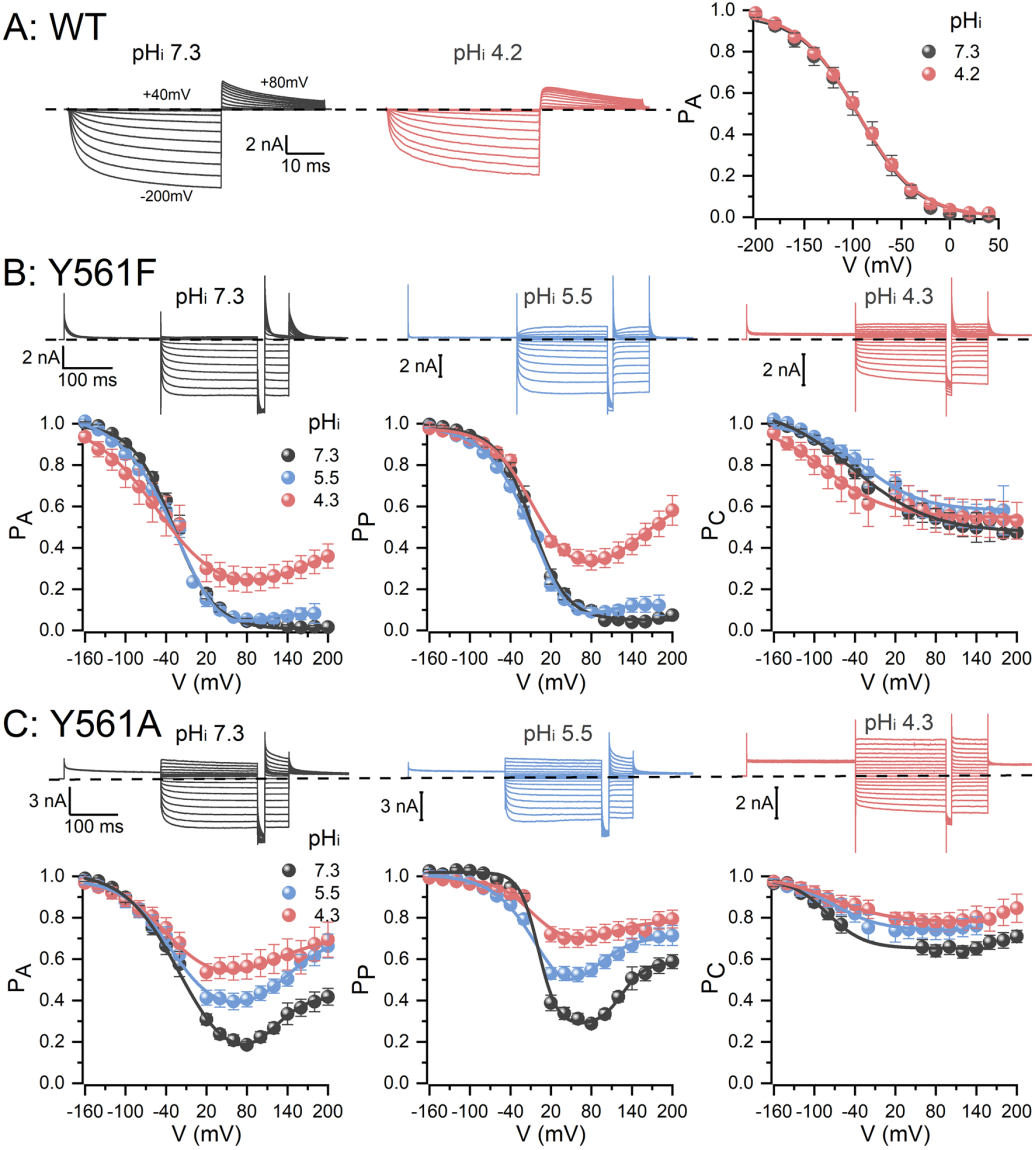
Y561F and Y561A CLC-2 mutant channels are sensitive to intracellular protons. **(A)** Intracellular acidification did not alter the voltage dependence of WT CLC-2 P_A_. Recordings like those shown on the left side were analysed to construct P_A_ vs V relations at pH_i_ of 7.3 (grey) and 4.2 (red). The voltage-dependent parameters V_0.5_ were -93.9 ± 7.9 mV and -93.4 ± 3.4 mV, at pH_i_ 7.3 and 4.2, respectively (n = 5). **(B,C)** Top panels. Cl^−^ currents from three different HEK293 cells expressing the Y561F **(B)** or Y561A **(C)** mutants were recorded in the presence of 140 mM intracellular Cl^−^ and intracellular pH 7.3 (grey), 5.5 (blue), 4.3 (red). The voltage protocol showed in Fig. 2A was utilized in these experiments. Extracellular pH and [Cl^−^]_o_ were 7.3 and 140 mM, respectively. **(B,C)** Bottom panels. Voltage dependence of P_A_, P_P_ and P_C_ for Y561F **(B)** and Y561A **(C)** are plotted using data collected at pH_i_ 7.3 (grey), 5.5 (blue) and 4.3 (red). Lines in **(A–C)** plots are fits of the data with a single (P_A_ in **A**; P_A_ and P_P_ at pH_i_ 7.3 and 5.5 and P_C_ in **B**; P_C_ in **C**) or double (P_A_ and P_P_ at pH_i_ 4.3 in **B**; P_A_ and P_P_ in **C**) Boltzmann equation used to calculate V_0.5_ and z parameters listed in Table 1.

Lowering pH_i_ from 7.3 to 4.2 did not affect the voltage-dependent activation of WT CLC-2 (Fig. 6A); this result emphasises the fact that intracellular H^+^ does not participate in WT CLC-2 activation. However, the effect of extracellular Cl^−^ on Y561A mutant suggests that E213 can be pushed inwardly by the Cl^−^ influx to induce channel re-opening. The opening of E213 in the inward direction could render it sensitive to intracellular H^+^. The Y561 mutant channels showed marked sensitivity to [H^+^]_i_ (Fig. 6B,C). Y561A channels showed larger currents than Y561F channels at positive potentials, after intracellular acidification (Fig. 6B,C). Decreasing pH_i_ from 5.5 to 4.3 shifted the P_A_ of both Y561F and Y561A by + 23 and + 7 mV, respectively (Table 1). Notably, the minimum Y561A P_A_ increased from 0.2 to nearly 0.6 after decreasing pH_i_ from 7.3 to 4.3 (Fig. 6C). Similar effects were observed in the voltage dependence of E213, described by P_P_, in the Y561F and Y561A channels (Fig. 6B,C, middle panels). At pH_i_ 4.3, E213 reopened in Y561F, whereas in Y561A it showed a substantial increment on the activation at pH_i_ 5.5 and 4.3. P_P_ reached a minimum value of about 0.7 at positive voltages indicating that E213 remains almost fully open all the time. Despite these results, the V_0.5_ of P_P_ measured in the negative range of voltages remained unchanged in both mutants (Table 1). Intracellular acidification also changed the magnitude and shifted the voltage dependence of the P_C_ towards negative voltages (Table 1). Taken together, these data show that intracellular acidification does not alter the voltage-gating of WT CLC-2, however, CLC-2 became sensitive to intracellular H^+^ once Y561 is mutated. We think that Y561 holds E213 in the closed position and shields it from intracellular H^+^ which would explain why intracellular acidification has no effects on WT CLC-2 gating^16,21,23^.

## Discussion

In this work, we demonstrate that both E213 and Y561 residues keep the CLC-2 chloride channel closed and we propose that permeant anions can open the gate by an electro-steric mechanism. The mutation analysis of the homology structure of CLC-2 suggested that a two-leaf gate formed by Y561-E213 closes the pore of CLC-2. Mutating either of these residues decreased the fractional contribution of the fast and slow components of gating, associated with the protopore and common gates, respectively. Also, the double mutant analysis showed that Y561 and E213 are energetically coupled. Thus, the gate of CLC-2 is formed by the interaction of the common and protopore gates, as reported for CLC-1^33^. Furthermore, these mutations facilitated the opening at negative potentials and the re-opening at positive voltages. We reasoned that E213 is decoupled from Y561 in the Y561A mutant channel, thus it is free to swing in the outward and inward directions guided by the Cl^−^ flux direction. This idea is supported by the experiments performed under low external Cl^−^ or high intracellular H^+^ concentrations. Our proposal that both common and protopore gate participate in CLC-2 gating is in agreement with previous findings showing that these gates are coupled in this channel^27^. Interestingly, in CLC-0 and CLC-1 the same pair of residues participate in voltage gating^33^ and pore and common gating mechanisms have been suggested to be coupled. However, despite that E and Y seems to be coupled by a hydrogen bond in CLC-0 and CLC-1, only common gating is altered by mutating the central Y^33^. The reason for this discrepancy with our results is unknown but it may reflect the intrinsic differences in voltage-dependent gating in CLC-0, CLC-1, and CLC-2 channels.

**Figure 7:**
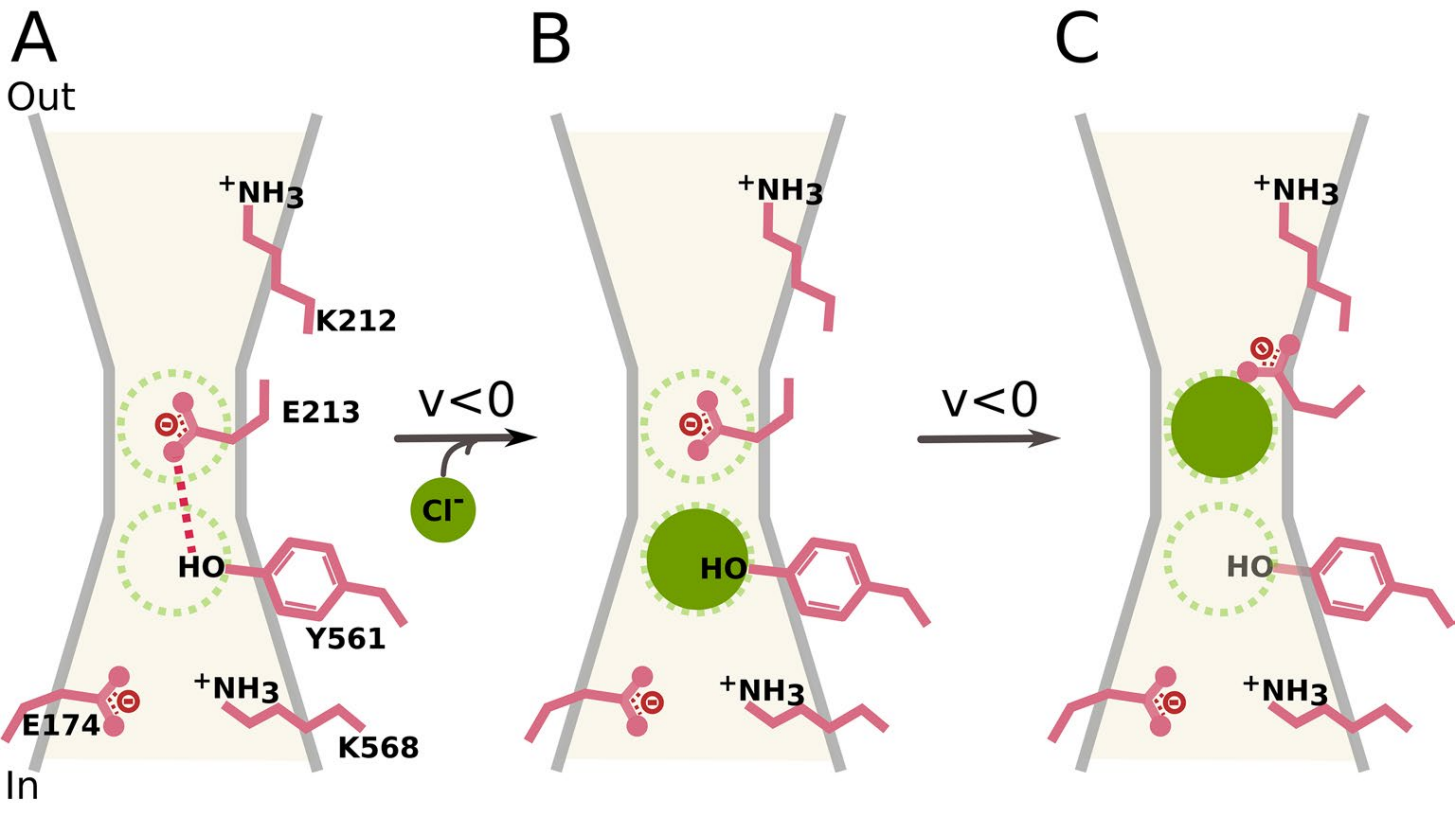
Schematic representation of the electro-steric activation of CLC-2. The Scheme depicts one pore and the side chains of E174, K568, Y561, E213 and K212 lining the pore. The empty pore remains closed by the Y561-E213 gate **(A)**. Upon a hyperpolarization (V < 0), intracellular Cl^−^ occupies the pore initiating the activation process **(B)**. The gate is splited by electro-steric repulsion and E213 adopts an outward-facing conformation thus coupling permeation to gating **(C)**.

Our results showed that permeant anions induce local conformational changes that are fundamental steps for gate-opening. In the closed state, E213 and Y561 are coupled (Fig. 7A). During the permeation process, the pore is occupied by more than one permeant anion. Permeating Cl^−^ ions encounter the Y561-E213 gate and split it by electro-steric repulsion (Fig. 7B). In the WT channel, permeant Cl^−^ repels E213 outwardly forcing E213 to adopt an outward-facing conformation (Fig. 7C). The separation of these residues by Cl^−^ effectively coupled anion permeation to pore gating to then enable anion permeation. We think that the Y561-E213 gate operates as a one-way check valve as has been proposed for K2P potassium channels^8^. E213 moves outwardly whereas Y561 prevents E213 from opening in the inward direction and shielding E213 from intracellular H^+^.

The structures of CLC Cl^−^/H^+^ exchangers and hCLC-1 show a pore that splits into two pathways at the cytosolic end. The canonical pathway transports Cl^−^ whereas the alternative pathway serves as an entryway for H^+^ to reach the glutamate gate^39^. The homology structure of CLC-2 shows an alternative pathway disconnected from the canonical pore. Thus, if the alternative water pathway serves as the H^+^ pathway but remains unplugged from the main pore, then E213 would not be protonated as we showed^40^.

A recent molecular dynamics simulation study of a CLC-2 homology structure reported that CLC-2 pore gating could indeed be Cl^–^dependent^32^. The authors used Markov state modelling and MD simulation of a homology structure of rat CLC-2. They proposed that entry of intracellular Cl^−^ requires rotation of the S168GI170 backbones and opening of E213 follows this step in a Cl^–^dependent manner. However, they suggest that Y561 (Y559 in their homology structure) is irrelevant for CLC-2 gating. We agree with the Cl^−^ dependence of CLC-2 gating, however, we have found that Y561 residue is critical for CLC-2 gating. Our mutational analysis demonstrated that Y561 holds E213 in the closed position and hinders E213 protonation. One possible explanation for this discrepancy would be the use of only one subunit to perform MD simulations. Experimental results suggest that the fast and slow gates are coupled in CLC-2^27,41^. Thus, residues forming the pore or residues participating in gating could have different conformational states in a homodimer than in a monomer. Also, the MD data were collected at 0 mV. At this voltage, the open probability of the gate is almost zero, which decreases the likelihood of observing conformational changes associated with gating.

We consider that an electro-steric gating mechanism might occur in other channels. For example, the voltage sensor-less K2P K^+^ channels display voltage dependence due to K^+^ ions movement along the pore^8,42^. Similarly, the voltage-dependent gating of viral K^+^ channel Kcv_NTS_ could rely on occupation by K^+^ of an external site^43^. Also, the outward movement of ions could explain the activation of PIEZO channels by voltage alone^44^. CLC-0 and CLC-1 show AMF behaviour, permeant anions facilitate gating, and non-permeant anions support voltage-dependent gating in CLC-1^18,19,22,24,25,45^ and the glutamate gate-less CLC-K is endowed with voltage-dependent gating after introducing a pore glutamate^46^. Thus, coupling ion permeation to pore gating in ionic channels might be more common than previously anticipated. Additional experiments combining functional, mutagenesis and structural analysis are needed to fully understand the gating coupled to the permeation.

## Methods

### Homology models of mouse CLC-2 and preparation of membrane-protein ensembles

We constructed the CLC-2^CLC-K^ model for the structure of the CLC-2 Cl^−^ channel (908 aa) using the I-Tasser^47^ server (https://zhanglab.ccmb.med.umich.edu/I-TASSER/) and the bovine CLC-K structure (5TQQ; Uniprot: E1B792; 687 aa) solved at 3.76 Å using cryo-EM^14^. Additional models, termed CLC-2^CLC-1^, were built using as templates the hCLC-1 structures determined by cryo-EM (6COY, 6QV6, 6QVB, 6QVU; UniProt: P35523; 988 aa)^13^, later available. hCLC-1 structures are truncated versions (469–626 residues by monomer) solved at 3.36 Å (6COY), 4.34 Å (6QVB), 3.63 Å (6QV6), and 4.20 Å (6QVU).

The protonation state of ionisable residues at pH 7.3 was determined by PROPKA^48^. The structures were embedded in symmetric bilayers of 1,2-dimyristoyl-sn-glycerol-3-phosphocholine (DMPC) or 1-palmitoyl-2-oleoyl-sn-glycero-3-phosphocholine (POPC) generated using CHARMM-GUI^49^ membrane builder (http://www.charmm-gui.org) and oriented using the Positioning of Proteins in Membrane server (PPM, http://opm.phar.umich.edu/server.php). The structures were solvated with water modelled by TIP3^50^ and 140 mM NaCl. The ensembles containing CLC-2^CLC-K^ and CLC-2^CLC-1^ consisted of 785 DMPC, 102741 TIP3, 800 Na^+^ and 416 Cl^−^ contained in a 170 × 170 × 158 Å^3^ simulation box, and 554 POPC, 43283 TIP3, 110 Na^+^, and 130 Cl^−^ contained in a 176 × 171 × 100 Å^3^ box, respectively.

### Cell culture, transient expression, and electrophysiological recordings

The study was carried out in compliance with the ARRIVE guidelines (https://arriveguidelines.org/). Wild type and mutant mouse CLC-2 channels were expressed in HEK-293 and *X. oocytes* and the macroscopic currents were recorded using the patch clamp and cut-open oocyte voltage clamp, respectively, as previously described^15–17,27,51,52^. WT or mutant DNAs were inserted in pIRES-II (for HEK-293 expression) o pGEM-T vectors (for *X. laevis* expression). HEK-293 cells were transiently transfected with 0.5 μg/μl of DNA using lipofectamine (Qiagen Inc., Valencia, CA, USA) following the manufacturer’s instructions. Mature *X. laevis* frogs, purchased from Aquanimals SA de CV, Querétaro, Mexico, were used to isolate oocytes via survival surgery. Frogs were care in accordance with Norma Oficial Mexicana NOM-062-ZOO-1999 and with guidelines of the Institutional Committee for Care and Use of Laboratory Animals from University Centre for Exact and Engineering Sciences of the University of Guadalajara (Comité Institucional del Cuidado y Uso de Animales en el Laboratorio CICUAL-CUCEI-UDG). The Institutional Committee for Care and Use of Laboratory Animals from University Centre for Exact and Engineering Sciences of the University of Guadalajara approved the frog oocytes isolation method and the cut oocytes electrophysiology experiments. Frogs were anesthetized with 0.1% tricaine (3-aminobenzoic acid ethyl ester) and a small portion of the ovary lobes containing oocytes was extracted. Oocytes were isolated under mechanical agitation and with the treatment of collagenase type II (Worthington Biochemical Corp., NJ, USA). Each oocyte was injected with 40 ng of RNA in-vitro transcribed using the T7 promoter mMESSAGE cRNA kit (Ambion, Austin, TX., USA) and DNA linearized with PmeI enzyme (New England Biolabs, Inc., Ipswich, MA, USA). Oocytes were incubated for 2–7 days at 17 °C in a standard oocyte saline solution. For patch-clamp, external and internal solutions contained (in mM): TEA-Cl 139, CaCl_2_ 0.5, HEPES 20 and D-mannitol 100; and, TEA-Cl 140, HEPES 20 and EGTA 20, respectively, and the pH was adjusted to 7.3 with TEA-OH. HEPES was substituted by MES to prepare solutions with low pH. The average tonicity of external and internal solutions was 387.9 ± 1.9 and 347.3 ± 2.6 mOsm/kg, respectively. For whole cell recordings, cells were held at 0 mV, followed by voltages steps to vary the membrane potential between + 60 or + 200 to − 160 mV in 20 mV increments and then repolarizing to + 60 or + 80 mV. Currents were recorded using pCLAMP 8 or 10 and a sampling rate of 500 kHz. For cut open oocyte recordings, the external and internal solutions contained (in mM): 130 NMDG-HCl, 4 MgCl_2_, 1 BaCl_2_ and 10 HEPES; and 136 NMDG (N-methyl-D-glucamine)-HCl, 2 MgCl_2_, 10 EGTA, 10 HEPES, respectively, and the pH was adjusted to 7.3 with NMDG. Internal solutions with different SCN^−^ or acetate mole fractions were prepared by mixing solutions containing 100% Cl^−^ with 100% SCN^−^ or acetate. Currents were filtered at 10 kHz and digitized at 100 kHz. All experiments were performed at room temperature (21–23 °C). Mutations were performed using standard PCR techniques and confirmed by sequencing. All chemicals were purchased from Sigma-Aldrich (St. Louis, MO, USA).

### Analysis

Current recordings were analysed with Clampfit or Analysis (UCLA, Los Angeles, CA, USA). Membrane and reversal potentials (V, E_r_) were corrected off-line using measured liquid junction potentials^53^. Recordings were analysed if E_r_ was near Cl^−^ Nernst potential. W_P_ and W_C_ (fractional contribution of the fast and slow components) and τ_f_ and τ_s_ (fast and slow time constants) were calculated by fitting the whole cell currents with a double exponential function:

2 $$ {{I}_{Cl}}={A}_{1}\left(1-{exp}^{\frac{{-t}}{{\tau_{f}}} }\right)+{{A}_{2}}\left(1-{exp}^{\frac {{-t}}{{\tau }_{s}}}\right)+{A}_{0} $$

where ${W}_{P}={A}_{1}/({A}_{0}+{A}_{1}+{A}_{2})$, ${W}_{C}={A}_{2}/({A}_{0}+{A}_{1}+{A}_{2})$, and ${W}_{cons}={A}_{0}/({A}_{0}+{A}_{1}+{A}_{2})$. E_r_ was determined from instantaneous I_Cl_-V plots. Conductance (G) at each V was calculated as I_Cl_/(V − E_r_). The open probabilities of the pore (P_p_) and common (P_C_) gates were calculated as described before^27^. Briefly, the time constant of the pore gate is ≤ 8 ms, thus, a 15 ms hyperpolarization to − 200 mV drives it into the fully open state (*P*_*p*_ ~ 1). Thus, we intercalated a 15 ms/− 200 mV pulse in test V after the current reached its steady state. The ratio of the steady-state current (= i * N_*T*_ * Pp * Pc where *P*_*p*_ * P_*C*_ = *P*_*A*_*, i* is the single-channel current and *N*_*T*_ is the total number of channels) to the current immediately after the 15/− 200 mV pulse (= i * N_T_* Pc), both sampled at the same V, is equal to P_p_. This protocol was applied at different V test to obtain *P*_*p*_ as a function of V. *P*_*C*_ was calculated as *P*_*A*_*/P*_*P*_ with P_A_ (= *G/G*_*max*_) determined in the same cell. The maximum conductance (G_max_) was estimated before normalization by fitting G–V curves with a single term Boltzmann equation. We determined the V dependence from fits of P_A_–V, P_P_–V or P_C_–V curves with a single or two terms Boltzmann equation (Eq. 3):

3 $$\frac{1}{1+{exp}^{-\frac{{z}_{O}F}{RT}(V-{V}_{0.5,O})}}+\frac{1}{1+{exp}^{-\frac{{z}_{rO}F}{RT}(V-{V}_{0.5,rO})}}$$

where z_O_ and z_rO_ are the apparent charge of the opening at negative V and re-opening at positive V, respectively, F is the Faraday constant, R is the gas constant, T is the temperature, and V_0.5,O_ and V_0.5,rO_ are the V needed to reach half value of its sigmoid effects. Data are plotted as mean ± SEM of n (number of independent experiments). Figures and fits were done using Origin (Origin Lab, Northampton, MA). Dashed black lines in Figures indicate I_Cl_ = 0.

Non-stationary noise analysis was performed as we described previously^54^. Shortly, WT ClC-2 and Y561A mutant currents were recorded at 100 and -100 mV repetitively (50–100 times) during 200 ms. The time-dependent variance (${\sigma }^{2}$)^55^:

4 $$ {\sigma}^{2}\left(t\right)=\frac{1}{2(M-1)}\sum_{j=1}^{M-1}{\left[{I}_{j+1}\left(t\right)-{I}_{j}(t)\right]}^{2} $$

where *M* is the total number of traces and ${I}_{j+1}$ and ${I}_{j}$ are two consecutive currents. The total number of channels (*N*) and single current (i) were determined by fitting the σ^2^ vs mean current (*Ī*) relationship with the equation^38^:

5 $${\sigma }^{2}=\left(1+{P}_{P}\right)\bullet i\bullet \stackrel{-}{I}-\frac{{\stackrel{-}{I}}^{2}}{N}$$

${P}_{P}$ is the open probability of the pore. For WT CLC-2 ${P}_{P}=0$ at + 100 mV, and ${P}_{P}=1$ in Y561A mutant at − 100 mV.

A one-way ANOVA with a Tukey post hoc test (p < 0.01) was used to test the statistical differences between the mean values of the voltage-dependent parameters. Significant differences are indicated by an asterisk, crosses or double dagger.

## Supplementary Information

Supplementary Figures.
