# Supplementary material for: Electro-steric opening of the clc-2 chloride channel gate
Source: Sci Rep. 2021 Jun 23;11:13127. doi: 10.1038/s41598-021-92247-3 (PMC8222222; doi:10.1038/s41598-021-92247-3)
Supplement: Supplementary file 1 — Supplementary Figures. [file 41598_2021_92247_MOESM1_ESM.docx]

Supplementary Information

Electro-Steric Opening of the CLC-2 Chloride Channel Gate

José J. De Jesús-Pérez^1^, G. Arlette Méndez-Maldonado^2^, Ana E. López-Romero^1^, David Esparza-Jasso^1^, Irma L. González-Hernández^2^, Víctor De la Rosa^3^, Roberto Gastélum-Garibaldi^2^, Jorge E. Sánchez-Rodríguez^2^, and Jorge Arreola^1,*^

^1^Physics Institute, Universidad Autónoma de San Luis Potosí, Ave. Dr. Manuel Nava #6, San Luis Potosí, SLP 78290, México.

^2^ Departamento de Física, Centro Universitario de Ciencias Exactas e Ingenierías, Universidad de Guadalajara. Blvd. M. García Barragán #1421, Guadalajara, Jal 44430, México.

^3^CONACYT. School of Medicine of Universidad Autónoma de San Luis Potosí. Ave. V. Carranza 2005, Los Filtros San Luis Potosí, SLP 78290, SLP, México.





1. **Block of WT and Y561A CLC-2 by Zn^2+^.** A: Currents generated by activation of WT CLC-2 were blocked by 0.5 mM extracellular Zn^2+^ (n=5). B: Currents generated by activation of Y561A CLC-2 mutant were blocked by 5 mM extracellular Zn^2+^ (n=7). Channels were activated using a -200 to +200 mV ramp.





1. **Functional characterization of WT, Y561F and Y561A chloride channels using the cut-open oocyte voltage clamp methodology.**
2. Chloride currents were recorded (colour-coded) from three different oocytes expressing WT CLC-2, Y561F CLC-2 and Y561A CLC-2 channels. Chloride currents were elicited by hyperpolarization steps from 40 (or 80) to -140 mV (or -120) in 10 mV steps followed by a single depolarization to 40 (or 60) mV to record tail currents (upper left). Immediately after, a 10 ms pulse to -160 mV was delivered followed by short pulse that varied the membrane voltage between 60 to -140 mV. The interpulse forced the probability of the pore gate *≈* 1 to calculate P_P_ and P_C_ as described in Methods. Currents were assessed at pH_i_ = pH_o_ = 7.3 and [Cl^-^]_i_ = [Cl^-^]_o_ = 140 mM. Dash line indicates I_Cl_ = 0.
3. Apparent open probability (P_A_) *vs* voltage.
4. Open probability of the pore gate (P_P_) *vs* voltage.
5. Open probability of the common gate (P_C_) *vs* voltage.

Open probabilities for WT, Y561F and Y561A CLC-2 channels (colour-coded) were determined as described in the methods section. Continuous lines represent fits with one or two terms Boltzmann (Equation 3) applied to determine the voltage-dependent parameters (V_0.5_ and z) listed in Table 1.
